# Invasive candidiasis and candidemia in pediatric and neonatal patients: A review of current guidelines

**DOI:** 10.18502/cmm.4.3.173

**Published:** 2018-09

**Authors:** Eleni Vasileiou, Athanasia Apsemidou, Timoleon-Achilleas Vyzantiadis, Athanasios Tragiannidis

**Affiliations:** 1 Hematology Oncology Unit, Pediatric Department of Aristotle University of Thessaloniki, AHEPA General Hospital, Thessaloniki, Greece; 2 Department of Internal Medicine, Papanikolaou General Hospital, Thessaloniki, Greece; 3 Department of Microbiology, Medical School, Aristotle University of Thessaloniki, Thessaloniki, Greece; 4 Hematology Oncology Unit, Department of Pediatric, Aristotle University of Thessaloniki, AHEPA General Hospital, Thessaloniki, Greece

**Keywords:** Invasive candidiasis, Candidemia, Children, Guidelines, Neonates, Prevention, Treatment

## Abstract

Several international and national guidelines have been proposed for the treatment and prevention of invasive candidiasis/candidemia (IC/C) in both neonatal and pediatric patients. This article is a review of the current guidelines, recommendations, and expert panel consensus of a number of associations and conferences on the prevention and management of IC and candidemia in both pediatric and neonatal patients. The investigated resources included the Infectious Diseases Society of America, the European Conference on Infection in Leukaemia, the European Society of Clinical Microbiology and Infectious Diseases, the German Speaking Mycological Society/Paul-Ehrlich Society for Chemotherapy, as well as the Canadian, Middle Eastern, and Australian guidelines. Echinocandins and liposomal amphotericin B (L-AmB) are the first-line agents in the treatment of IC and candidemia both for immunocompetent and immunocompromised pediatric patients. The recommendations suggested to keep patients under sterile conditions for at least 14 days after blood cultures as the prompt initiation of antifungal treatment. Guidelines addressing the neonates recommended to use L-AmB, deoxycholate AmB (D-AmB), and fluconazole based on three main principles of no previous exposure to azoles, the prompt initiation of antifungal treatment, and control of predisposing underlying conditions. Despite minor differences among the investigated guidelines, general treatment recommendations suggest the prompt initiation of antifungal treatment and control of all predisposing underlying conditions.

## Introduction

Invasive candidiasis and candidemia (IC/C) are important causes of morbidity and mortality, mainly in immunocompromised and hospi-talized patients [1-8]. More than 750,000 cases of invasive candidiasis are annually reported worldwide [9]. The IC/C mainly affect the immune-compromised children, as well as the pediatric and neonatal patients admitted to intensive care units (ICUs). The most common species of *Candida*, isolated from patients, are *C. albicans*, *C. glabrata*, *C. tropicalis*, *C. Parapsilosis*, and *C. krusei* [10-13]. 

Nowadays, multiple pharmacotherapeutic options are available for the treatments of IC/C, such as older and newer formulations of AmB and triazoles, as well as echinocandins [2]. Despite improvements in disease prevention, IC/C account for high mortality rates ranging 36-63%. However, regarding the pediatric patients, this rate has been reported to have a range of 7.7-26% in various studies [14-21].

This article involved the description and comparison of the international and national guidelines on IC/C treatment and prevention published during the last 8 years. To this end, we reviewed recommendations and guidelines issued by international medical societies and expert panels in the United States of America, Europe, Germany, Canada, the Middle East, and Australia. The most recent guidelines were published in 2016 by the Infectious Diseases Society of America (IDSA) [22] and the European Conference on Infections in Leukemia (ECIL-6) [23], followed by Australian consensus guidelines (2014), Middle Eastern (2013), the European Society for Clinical Microbiology and Infectious Diseases (ESCMID; 2012), German Speaking Mycological Society/Paul-Ehrlich Society for Chemotherapy (DMYKG/PEG; 2011), and Canadian clinical practice (2010) [24-28]. 

In the above-mentioned guidelines, patients were divided into four major groups, namely neutropenic or immunocompromised, non-neutropenic or immune-competent, ICU patients, and neonates. It should be mentioned that only ESCMID has issued recommendations exclusively for pediatric patients with *Candida *spp. infections. On the other hand, ECIL group published guidelines for the diagnosis, prevention, and management of invasive opportunistic fungal diseases (IFDs), which was not exclusive to IC/C in pediatric patients with hematologic malignancies or hemato-poietic stem cell transplantation (HSCT) recipients. 

The IDSA guidelines published in 2016 addressed adults; however, it recommended to use the same therapeutic approach for the pediatric patients while considering the drug dosage and pharmacokinetics. The DMYKG/PEG and Australian group guidelines provided detailed recommendations for antifungal treatment and dosage in pediatric and neonatal population. On the other hand, the Canadian and Middle East group recommendations focused on the adult population. 

The strength of recommendation and the quality of evidence vary between different working groups. In general, the recommendations are assessed by using a scoring system proposed by the IDSA. In this scoring system, the strength of the recommendations is rated on a 5-level scale (A: strongly recommended, B: generally recommended, C: optional, D: generally not recommended, and E: never recommended). Furthermore, the quality of the evidence for a recommendation is evaluated on a 3-level scale (I: evidence from ≥1 properly randomized, controlled trial, II: evidence from ≥1 well-designed clinical trial without randomization, cohort or case-control analytical studies [preferably from >1 center], and multiple time-series, or dramatic results from uncontrolled experiments, and III: evidence from the opinions of respected authorities, based on clinical experience, descriptive studies, or reports of expert committees). It should be noted that the important differences for pediatric patients were considered in this scoring system. 

***Treatment of invasive candidiasis and candidemia in children***

Regarding the choice of antifungal agent and disease management, the applied rules to adult patients are similar to those utilized for children. These similarities include the prompt initiation of antifungal treatment, removal of central venous catheter (CVC, if considered), control of predisposing underlying conditions, appropriate choice of an anti-*Candida* agent or the of combined antifungal therapy in certain circumstances, clinical assessment of deep tissue infection, and continuation of therapy in case of uncomplicated candidemia 14 days after blood cultures. These recommendations should be implemented under sterile conditions where there are no clinical symptoms of the infections [24]. 

According to the ESCMID guidelines, echinocandins (i.e., caspofungin [A-I] with a loading dose of 70 mg/m^2^, followed by 50 mg/m^2^/day, 2-4 mg/kg/day micafungin [A-I], and anidulafungin [B-II] with a loading dose of 3 mg/kg, followed by 1.5 mg/kg/day) are the alternative agents for the treatment of IC/C in children [24]. Caspofungin and micafungin are approved by both Food and Drug Administration and European Medicine Agency for pediatric patients, while anidulafungin is assigned for IC treatment in adult patients. 

Additionally, 3 mg/kg/day liposomal AmB (A-I) is an alternative first-line agent. Moreover, 0.6-1 mg/kg D-AmB (C-I) can be used for IC/C in children. However, it is graded lower than lipid preparations due to having less favorable toxicity profile. Triazoles should be considered for hemodynamically stable patients in institutions with a low incidence of less susceptible *Candida* spp. Voriconazole (B-I) is recommended for IC because it shows more potency than fluconazole, especially in infections caused by *C. glabrata *and *C. krusei* [24].

The IDSA recommends echinocandin (i.e., caspofungin [A-I] with a loading dose of 70 mg/m^2^, followed by 50 mg/m^2^/day, 2 mg/kg/day micafungin [A-I] that can be increased to 4 mg/kg/day in children weighing <40 kg, and 1.5 mg/kg/day anidulafungin [A-I]) as the first-line pharmaceutical agent in non-neutropenic patients. The L-AmB (A-I) can be administered as an alternative; however, its possible toxicity should be considered. 

Fluconazole is only recommended in non-neutropenic patients who are not critically ill and have no prior azole exposure (A-I). Transition from AmB to fluconazole is recommended after 5-7 days in patients who are susceptible to fluconazole isolates and clinically stable with negative blood cultures. Echinocandins (A-II) and L-AmB (A-II) are suggested to be used in neutropenic patients [22].

Similar to the above-mentioned guidelines, the ECIL-6 expert panel recommends echinocandins (B-II) as the fist-line choice for IC/C before species identification and L-AmB (B-II) as an alternative option. The absence of a strong recommendation is attributed to the sparsity of data for granulocytopenic patients and tissue-invasive disease. Treatment with fluconazole is not recommended for infections by *C. krusei *and *C. glabrata*, due to the high resistance of isolates*. *It is worth mentioning that combination antifungal chemotherapy should be considered in special circumstances, such as a severe life-threatening infection [23].

The DMYKG/PEG society recommendations support the use of L-AMB (A-I) and echinocandin (i.e., caspofungin [A-II] and micafungin [A-I]) [25]. Canadian, Australian, and Middle Eastern guidelines also suggest echinocandins and L-AmB as the options in the treatment of pediatric patients with IC/C [26-28].

The ECIL-6 group proposes the removal of CVC or other prosthetic devices in all cases of candidemia. On the other hand, IDSA introduces gastrointestinal tract rather than CVCs as the main source of candidemia in neutropenic patients; therefore, it suggests to decide on CVC removal based on each patient’s condition [22, 23]. Finally, IDSA weakly recommends the use of granulocyte colony-stimulating factor (G-CSF) in neutropenic patients with persistent candidemia [22]. Table 1 tabulates the recommendations for the treatment of IC/C in children.

*Empiric treatment for suspected invasive candidiasis and candidemia*

Empiric antifungal therapy is considered as the standard of care in critically ill patients with several risk factors for invasive candidiasis. The ESCMID guidelines address the issue of empiric antifungal therapy in haemato-oncological patients with prolonged neutropenia and refractory fever despite the use of broad-spectrum antibiotics. This guideline strongly recommends to use L-AmB (A-I), 1-3 mg/kg, and caspofungin (A-I) with a loading dose of 70 mg/m^2^/day, followed by 50 mg/m^2^/day. Fluconazole (B-II) and D-AmB (B-II) are recommended as second-line agents. No data exist for non-neonatal pediatric patients in ICU [24].

The IDSA highly recommends the initiation of empiric antifungal treatment in critically ill non-neutropenic patients in ICUs with risk factors for IC/C, clinical signs of septic shock, and no other known causes of fever. The agent of choice is echinocandin (A-II). However, fluconazole (A-II) with a loading dose of 800 mg (12 mg/kg), followed by 400 mg (6 mg/kg daily) or 3-5 mg/kg daily lipid formulation AmB (A-II), could be used alternatively if there is no intolerance or resistance [22].

Canadian guidelines strongly suggest empirical antifungal therapy for neutropenic patients with persistent fever after 4-7 days of broad-spectrum antibacterial therapy. Recommended drugs are L-AmB (A-I) and caspofungin (A-I). Furthermore, the D-AmB, fluconazole, and voriconazole could be used alternatively in some groups of patients [26]. 

*Prevention of invasive candidiasis and candidemia in children*

Primary prophylaxis should be considered as a treatment for patients at the high risk of developing IC/C. The incidence of IC in patients with acute myeloid leukemia, recurrent leukemia, and the subsequent allogeneic HSCT is estimated as 5-15%. The ESCMID guidelines strongly recommend prophylactic administration of fluconazole (A-I) at a dose of 8-12 mg/kg (once daily; intravenous [IV] or oral) or 1 mg/kg/day micafungin (A-I and A-II, respectively; IV) in allogeneic or autologous HSCT recipients and children with acute lymphoblastic, myeloid, or recurrent leukemia. 

In allogeneic HSCT recipients with the age of ≥ 2 years, 8 mg/kg and 9 mg/kg voriconazole (A-I) are used for intravenous and oral administrations, respectively. Further alternatives include 2.5 mg/kg itraconazole (B-II) every 12 h for patients older than 2 years and 200 mg posaconazole (B-II) administered orally three times a day for patients older than 13 years with allogeneic HSCT, acute myeloid, or recurrent leukemia [24].

**Table 1:** Comparison of recommendations on the prevention/therapy of invasive candidiasis/candidemia in children

	**ESCMID**	**IDSA**	**ECIL-6**	**DMYKG/PEG**	**Canada**	**Middle East**	**Australia**
**Treatment**	Caspofungin (A-I), Micafungin (A-I), Anidulafungin (B-II) L-AmB (A-I), Fluconazole (B-I), Voriconazole (B-I) D-AmB (C-I)	Caspofungin (A-I), Micafungin (A-I), Anidulafungin (A-I) L-AmB (A-I) Fluconazole (A-I) in non-neutropenic pts, Caspofungin (A-II), Micafungin (A-II), Anidulafungin (A-II) L-AmB (A-II) in neutropenic pts	Caspofungin (B-II), Micafungin (B-II), L-AmB (B-II), Fluconazole (B-II), Voriconazole (B-II) pts with hematological malignancies or HSCT recipients	Caspofungin (A-II) Micafungin (A-I), L-AmB (A-I), Fluconazole (A-II), Voriconazole (A-II)	Echinocandins (A-I) Fluconazole (A-I) non neutropenic pts L-AmB (A-I), Caspofungin (A-I), Micafungin (B-III), neutropenic pts	Echinocandins (A) L-AmB (A), Non neutropenic pts, Echinocandins (B) neutropenic pts	Most recommendations in children, unless otherwise stated, would be considered Grade C at best, due to the paucity of dedicated paediatric randomised data.
**Empiric therapy**	L-AmB (A-I) Caspofungin (A-I), Fluconazole (B-II), D-AmB, (B-II)	L-AmB (A-II), Echinocandins(A-II), Fluconazole (A-II),	L-AmB (A-I), Caspofungin (A-I),		L-AmB (A-I) Caspofungin (A-I) Fluconazole (B-II) D-AmB (B-II), Voriconazole (B-I)		
**Prevention**	Micafungin (A-I) Fluconazole (A-I), Voriconazole (A-I), Posaconazole (B-II), Itraconazole (B-II) in HSCT recipients, Micafungin (A-II) Fluconazole (A-I), Voriconazole (B-I), Posaconazole (B-II), Itraconazole (B-II) in AML and recurrent leukemias	Echinocandins (B-III) Fluconazole (B-II)	Fluconazole (A-I) Voriconazole (B-I), Itraconazole (B-I) and (C-I) for allogeneic HSCT recipients without and with GvHD, respectively		Fluconazole (A-I) Posaconazole (A-I) Itraconazole (B-I)		

D-AmB: deoxycholate amphotericin B

DMYG/PEG: German Speaking Mycological Society/Paul-Ehrlich Society for hemotherapy

ECIL-6: European Conference on Infections in Leukemia

ESCMID: European Society for Clinical Microbiology and Infectious Diseases

GvHD: graft-versus-host disease

HSCT: hematopoietic stem cell transplantation

IDSA: Infectious Diseases Society of America

L-AmB: liposomal amphotericin B

**Table 2:** Comparison of recommendations on the prevention/therapy of invasive candidiasis/candidemia in neonates

	**ESCMID**	**IDSA**	**DMYKG/PEG**
**Treatment**	D-AmB (B-II) L-AmB (B-II) Fluconazole (B-II) Micafungin (B-II)	D-AmB (A-II) L-AmB (C-III) Fluconazole (A-II)	L-AmB (A-II) Fluconazole (A-II) Micafungin (A-I) Caspofungin(A-II)
**Prevention**	Fluconazole (A-I) Nystatin (B-II) Lactoferrin ± Lactobacillus (B-II)	Fluconazole (A-II) Nystatin (C-II) Lactoferrin (C-II)	

D-AmB: deoxycholate amphotericin B

DMYG/PEG: German Speaking Mycological Society/Paul-Ehrlich Society for hemotherapy

ESCMID: European Society for Clinical Microbiology and Infectious Diseases

IDSA: Infectious Diseases Society of America

L-AmB: liposomal amphotericin B

The IDSA group weakly recommends the administration of fluconazole (B-II) or echinocandin (B-III) in high-risk patients in ICUs with a high rate of IC and noted to consider daily bathing with chlorhexidine [22]. Moreover, Canadian guidelines suggest antifungal prophylaxis in this group of patients. Canadian expert panels also recommend 400 mg/day oral fluconazole (A-I) or 200 mg posaconazole (A-I; three times a day) in patients with leukemia or HSCT recipients [26]. The ECIL group highly suggests the use of fluconazole (A-I) during the granulocytopenic phase before and after engraftment in the absence of graft-versus-host disease (GvHD), until the discontinuation of immunosuppressive therapy [23]. Table 1 presents the recommendations for the prevention of IC/C in children. 

*Treatment for neonatal candidiasis*

The IC/C is a common infection in premature neonates in neonatal intensive care units (NICUs). As a result, any premature newborn with clinical or laboratory evidence of IC/C should be assessed for disseminated disease. A unique manifestation of IC/C in neonates is hematogenous *Candida* meningoencephalitis (HCME) that can result in neurodevelopmental abnormalities. It is observed that the smaller the gestational age, the higher the incidence of IC/C. 

Unlike adults and older children, neonates tolerate D-AmB very well with no significant signs of nephrotoxicity. Moreover, the therapeutic concentrations of echinocandins are not easily achieved in the central nervous system (CNS) and the urine; therefore, higher doses are used in neonates. According to IDSA guidelines, the treatment approach of IC/C in neonates should include a lumbar puncture and a dilated retinal examination in the presence of positive blood or urine cultures and the removal of CVC. The optimal treatment duration recommended is 2 weeks after the clearance of *Candida* spp. from the bloodstream and resolution of the clinical and microbiological signs attributable to candidemia [22]. 

The ESCMID moderately suggests the use of 1 mg/kg/day D-AmB (B-II) with no specific clinical data for the treatment of HCME. It also marks that 2.5-7 mg/kg/day L-AmB (B-II) penetrates the CNS in a preclinical model of HCME and shows antifungal activity in the brain. Additionally, fluconazole (B-II) with a loading dose of 25 mg/kg, followed by 12 mg/kg, should be considered in neonates, who have not previously received this agent, with potential limitations. Echinocandins are increasingly used for these patients. Furthermore, 4-10 mg/kg/day micafungin (B-II) is recommended for the treatment of neonatal candidiasis. It is noted that there is a dose-dependent penetration of micafungin into the CNS, and that a higher dose (10 mg/kg/day) should be used if HCME is suspected [24]. 

On the other hand, IDSA strongly recommends 1 mg/kg/day D-AmB (A-II) for neonates with disseminated candidiasis and 12 mg/kg oral or IV fluconazole (A-II) for those who have not been on azole prophylaxis. In addition, 5 mg/kg/day L-AmB (C-III) is an alternative in neonates with limitations in the presence of urinary tract infection [22]. 

Finally, the DMYKG/PEG society gives a strong recommendation for the use of L-AMB (A-I), micafungin (A-I), caspofungin (A-II), and fluconazole (A-II), while the combination of L-AmB and flucytosine is undefined due to poor evidence (C-III) [25]. Table 2 summarizes the recommendations for the treatment of IC/C in neonates. 

*Prevention of neonatal candidiasis*

Antifungal prophylaxis should be taken into account, especially in extremely low-birth-weight premature neonates. The ESCMID moderately supports the use of nonabsorbable agents to decrease the *Candida* burden in the gastrointestinal tract and fungal translocation into the bloodstream. As a result, this group recommends the use of 1 ml of nystatin suspension (B-II; 100,000 U/ml) every 8 h, with the potential limitation of developing necrotizing enterocolitis and miconazole (D-II). 

The ESCMID experts suggest the use of 100 mg/day lactoferrin (B-II) alone or in combination with lactobacillus for neonates weighing < 1500 g for the reduction of late-onset sepsis, including the episodes attributed to *Candida* spp. In addition, ESCMID strongly recommends intravenous or oral administration of 3-6 mg/kg/dose fluconazole (A-I; twice weekly) for all neonates weighing < 1,000 g in NICUs with a high frequency of IC/C. Miconazole oral gel is contraindicated because its use may lead to the development of triazole resistance, which can subsequently render fluconazole useless [24].

The IDSA strongly recommends 3-6 mg/kg intravenous or oral fluconazole prophylaxis (A-I) twice weekly for 6 weeks in NICU-admitted neonates with the birth weights of < 1,000 g with high IC/C incidence. According to IDSA guidelines, oral nystatin (C-II) is only weakly recommended when fluconazole is not available or triazole resistance is suspected. Finally, it is mentioned that lactoferrin (C-II) could be used in neonates with the birth weight of < 1500 g; however, this medication is not available at the present in the US hospitals [22]. Table 2 presents the recommendations for the prevention of IC/C in children. 

## Conclusion

Despite minor differences among the investigated guidelines and recommendations, it is evident that echinocandins and L-AmB are the first-line pharmaceutical agents in the treatment of IC/C both for immunocompetent and immunocompromised pediatric and adult patients. Fluconazole and voriconazole are potential alternatives for first-line treatment in the overall population provided that there is hemodynamically stability and no previous exposure to azoles. 

Voriconazole is recommended for the treatment of IC by ESCMID and ECIL groups because it shows more potency than other azoles, especially in infections caused by *C. glabrata* and *C. krusei*. The ECIL-6 group suggests the removal of CVCs or other prosthetic devices in all cases of candidemia. However, IDSA emphasizes that in neutropenic patients, the main source of candidemia is not CVCs but the gastrointestinal tract. Therefore, this group recommended to decide on CVC removal based on the condition of each patient. 

Furthermore, all societies and expert panels agree that the minimum duration of therapy after which blood cultures are sterile is a 14-day period, although clinical evaluation of deep tissue infection should be considered and symptoms of candidemia are resolved. Empiric therapy of suspected IC/C with L-AmB or caspofungin is also recommended. Although there are differences among various societies in terms of the pediatric guidelines for the prevention of IC/C in high-risk patients, a number of agents, including fluconazole, voriconazole, and micafungin could be considered as possible treatments. 

Recommendations for the treatment of neonates with IC/C favor the use of L-AmB, D-AmB, and fluconazole in neonates with no previous exposure to azoles. Echinocandins are increasingly used in this population. Nystatin, fluconazole, and lactoferrin are considered acceptable agents for the prevention of neonatal candidiasis. Finally, in most of the cases, CVCs should be removed or replaced as soon as possible, and suspicion for the disseminated disease should be eliminated.
